# One-Step Hydrothermal/Solvothermal Preparation of Pt/TiO_2_: An Efficient Catalyst for Biobutanol Oxidation at Room Temperature

**DOI:** 10.3390/molecules29071450

**Published:** 2024-03-24

**Authors:** Lijun Lei, Qianyue Cao, Jiachen Ma, Fengxiao Hou

**Affiliations:** 1 School of Energy and Power Engineering, North University of China, Taiyuan 030051, China; neromjc@gmail.com (J.M.); mercatushou@outlook.com (F.H.); 2 School of Chemistry and Chemical Engineering, North University of China, Taiyuan 030051, China; caoqianyuedw@163.com

**Keywords:** biobutanol, catalytic oxidation, hydrothermal/solvothermal, size effect, Pt-TiO_2_

## Abstract

The selective oxidation of biobutanol to prepare butyric acid is an important conversion process, but the preparation of low-temperature and efficient catalysts for butanol oxidation is currently a bottleneck problem. In this work, we prepared Pt-TiO_2_ catalysts with different Pt particle sizes using a simple one-step hydrothermal/solvothermal method. Transmission electron microscopy and X-ray diffraction results showed that the average size of the Pt particles ranged from 1.1 nm to 8.7 nm. Among them, Pt-TiO_2_ with an average particle size of 3.6 nm exhibited the best catalytic performance for biobutanol. It was capable of almost completely converting butanol, even at room temperature (30 °C), with a 98.9% biobutanol conversion, 98.4% butyric acid selectivity, and a turnover frequency (TOF) of 36 h^−1^. Increasing the reaction temperature to 80 and 90 °C, the corresponding TOFs increased rapidly to 355 and 619 h^−1^. The relationship between the electronic structure of Pt and its oxidative performance suggests that the synergistic effect of the dual sites, Pt^0^ and Pt^2+^, could be the primary factor contributing to its elevated reactivity.

## 1. Introduction

Given the escalating levels of air pollution and the impacts of climate change, it is imperative to explore alternative sources of energy to replace fossil fuels in the production of fuels and chemicals. In recent years, biomass energy has gained substantial attention as a notable alternative energy option and the primary renewable carbon source. Researchers have shown significant interest in exploring the efficient utilization of biomass feedstocks and the conversion of biomass platform molecules, which have become prominent areas of research focus [1,2]. Several process routes have been designed and developed for biomass energy utilization. One of these pathways involves converting biomass feedstock into an aqueous solution of acetone–ethanol–butanol (ABE) through biofermentation [3]. Bioethanol has already made significant strides in substituting fossil fuels in North and South America [4]; however, biobutanol is emerging as a superior alternative to bioethanol as an oil additive. This is attributed to its insolubility in water, higher energy density, greater blending capacity with gasoline, and reduced corrosive impact on engines [5].

In addition to potential applications in fuels, the high-value conversion of biofermented alcohols into important chemical feedstocks is also an important route. The selective conversion of biobutanol and ethanol to organic acids is one of the most promising routes [3]. The current main synthesis process for butyric acid is the hydroformylation of propylene to obtain butyraldehyde, followed by further oxidation to obtain butyric acid. The synthesis of butyric acid by the one-step catalytic oxidation of an aqueous solution of butanol obtained from biomass is more sustainable and low-carbon than the current petroleum-based synthesis process [6]. In the realm of green chemistry, there is a significant emphasis placed on replacing harmful stoichiometric oxidants typically used in oxidation reactions with more accessible and cost-efficient alternatives like O_2_ or air. Furthermore, there is a movement towards substituting homogeneous metal complex catalysts with recyclable heterogeneous versions that exist in solid form [7]. At the same time, the biofermentation broth inherently includes water. The advancement of heterogeneous catalytic systems that can effectively oxidize alcohol in the aqueous phase is crucial for various applications of biobutanol and ethanol. These applications encompass the production of fragrances, downstream chemicals, and pharmaceutical intermediates, among others [1]. A variety of catalytic systems for alcohol oxidation based on different metal active components, such as Pt [8,9], Pd [10,11], Ru [12,13], and Au [14,15,16], have been reported. However, most of the catalysts have high catalytic activity for activated alcohols such as benzyl alcohol, furfuryl alcohol, etc., whereas for non-activated aliphatic alcohols, the catalytic activity of conventional catalysts is relatively low [7,17]. In current research, the reaction temperatures for activated alcohols [7,18,19] typically fall within the range of 25–90 °C. The alcohol-to-metal molar ratios in these reactions vary from 200 to 8000, and turnover frequencies (TOFs) typically range from 200 to 12,000 h^−1^. In contrast, reactions involving non-activated aliphatic alcohols [3,20,21,22,23] typically occur at temperatures between 55 and 160 °C. The alcohol-to-metal molar ratios in these reactions range from 10 to 2000, and the TOF values, which are roughly estimated based on the corresponding activity, generally fall between 2 and 500 h^−1^.

The oxidation of non-activated aliphatic alcohols can be enhanced by incorporating potent soluble bases like KOH, NaOH, or K_2_CO_3_. Nevertheless, this practice may lead to issues such as equipment corrosion and the production of highly alkaline waste solutions [22]. Some strategies have been used to improve the catalytic performance of aliphatic alcohols without the use of soluble strong bases. Dong et al. [24] prepared alkaline supports for the liquid-phase ethanol oxidation reaction by the high-temperature treatment of carbon nanotubes with KOH, and the catalytic activity of the alkaline-treated catalysts was four times higher than that of the untreated ones. The leaching of the basic sites was not significant after two cycles of the catalysts. Wang [25] and Oberhanser [23] and their co-workers complexed pyrrolidone and diamine with metal particles to enhance the catalytic activity of alcohols, respectively. The ligand modification increased the alcohol oxidation activity by more than 30%. Additionally, the coordination between the nitrogen-containing ligands and the metal particles prevented the loss of the ligand molecules. Furthermore, Gandarias et al. [3] prepared PtPd bimetallic catalysts to improve butanol oxidation performance, and the reported butanol catalytic oxidation activities of PtPd(1:1)/TiO_2_ were 1.8 and 3.4 times higher than the activities of the monometallic Pt/TiO_2_ and Pd/TiO_2_, respectively, showing good synergistic catalytic effects for the PtPd bimetallic catalysts. Despite the many efforts made by researchers to design low-temperature and efficient catalysts for alcohol oxidation, only Pt/ZnO and Pt/Ca-ZSM-5 have been reported to be able to convert activated benzyl alcohol to benzaldehyde at room temperature close to completely [26,27]. However, to the best of our knowledge, few catalysts are currently available to efficiently catalyze the selective oxidation process of non-activated alcohols at room temperature (≤30 °C) without a base and UV illumination (more than 50% conversion) in reported catalysts.

Furthermore, the activity of alcohol oxidation is closely related to the size and electronic structure of the active metal species [28]. Metal nanoparticles are nanocrystals with sizes ranging from 1 to 100 nm. Compared to clusters and monoatomic metal species, the high structural stability and simplicity of preparation of nanoparticles continue to be areas of great interest for researchers. Currently, commonly used catalyst preparation methods, such as impregnation, deposition precipitation, and sol–gel methods, make it challenging to precisely control the size and electronic structure of metals [29]. One of the effective methods is the solution-phase colloidal method. The modulation of metal particle size is achieved by controlling the nucleation and growth processes of metal species through the addition of chelating and stabilizing agents [29,30]. In addition, atomic layer deposition (ALD) has been widely used in recent years to prepare metal catalysts loaded with various size distributions [31]. However, the former uses more expensive chelating agents and stabilizers, as the subsequent heat treatment of the catalyst to remove these organics leads to the sintering of metal species, while the limited capacity and the need for organometallic precursors with low boiling points restrict the widespread application of ALD.

In the present work, Pt-TiO_2_ with different Pt sizes (from 1.1 nm to 8.7 nm) was controllably prepared using a one-step hydrothermal/solvothermal method. Among the different catalysts, Pt(3.6)-TiO_2_ exhibited excellent aliphatic alcohol oxidation activity and could efficiently convert biobutanol to butyric acid at room temperature (30 °C) with a yield of 97.3% and a TOF of 36 h^−1^, showing high low-temperature oxidation performance for butanol. Various characterization measurements and activity tests were performed to reveal the effect of Pt particle size on butanol oxidation performance.

## 2. Results and Discuss

### 2.1. Characterization of the Pt-TiO_2_ Catalysts

**Figure 1:**
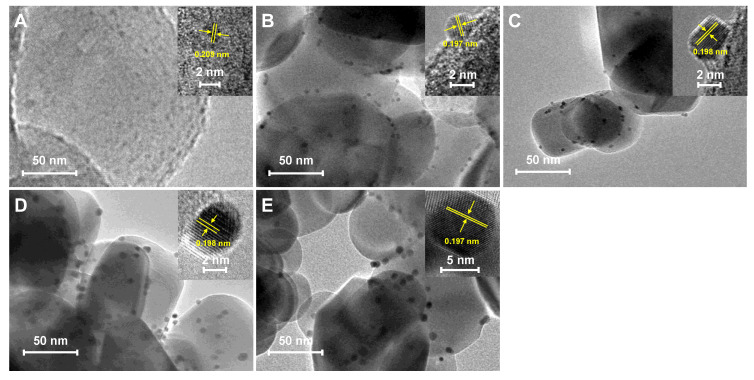
TEM images of Pt-TiO_2_ with different Pt particle sizes; the insets are the high-resolution TEM images. (**A**) Pt(1.1)-TiO_2_; (**B**) Pt(2.6)-TiO_2_; (**C**) Pt(3.6)-TiO_2_; (**D**) Pt(5.3)-TiO_2_; (**E**) Pt(8.7)-TiO_2_.

Figure 1 displays the TEM images and corresponding high-resolution transmission electron microscopy (HR-TEM) images of the Pt-TiO_2_ catalysts, exhibiting different particle sizes of Pt (insets in the images). The actual loading of Pt for all Pt-TiO_2_ samples measured by ICP-OES is shown in Table S1. It is evident that the Pt particles in Pt-TiO_2_ catalysts tend to be more dispersed at the edges of the TiO_2_ support. Through meticulous examination and measurement of the HR-TEM images of the Pt-TiO_2_ sample with a Pt size of 1.1 nm, it was determined that the spacing between the lattice stripes was 0.209 nm. This corresponds to the crystalline surface of PtO_2_ (002) (#97-007-6431). For other Pt-TiO_2_ samples with Pt sizes of 2.6, 3.6, 5.3, and 8.7 nm, the lattice stripes predominantly ranged from 0.197 to 0.198 nm. This spacing corresponds to the lattice stripes observed on the Pt (200) crystalline surface (PDF#97-018-0981). This indicated that the Pt particles were successfully loaded on the TiO_2_ surface [32]. A total of 10 to 30 representative TEM images of Pt-TiO_2_ were carefully chosen to analyze the Pt particle size. The results, as depicted in Figure S1, revealed significant variations in the particle size among the Pt-TiO_2_ catalysts prepared using five different solvent hydrothermal/solvothermal methods. Notably, the catalysts synthesized solvothermally in a dilute ammonia aqueous solution exhibited an average Pt size of merely 1.1 nm. Conversely, when the solvent was changed from water to methanol, ethanol, and ethylene glycol, the corresponding Pt particle sizes progressively increased to 2.6, 3.6, 5.3, and 8.7 nm, respectively. The variations in the nucleation and growth processes of Pt particles during the synthesis process may be attributed to the differences in the solubility of H_2_PtCl_6_ in various solvents. Compared to the hydrothermal method, the Pt particles prepared by the solvothermal method with dilute ammonia as the solvent were smaller in size (only 1.1 nm). This may be attributed to the strong coordination between ammonia and Pt, resulting in a higher dispersion of the prepared Pt species, in agreement with the literature [33]. When organic solvents like methanol, ethanol, and ethylene glycol were used for the solvothermal process, the obtained Pt particle sizes were all larger than the hydrothermal process. Compared to water, these alcohols are reductive and allow for rapid nucleation and growth processes in Pt nanocrystals. The differences in solubility of H_2_PtCl_6_ in methanol, ethanol, and ethylene glycol and the reducing properties of the three alcohols in turn led to the preparation of three Pt-TiO_2_ catalysts affording different Pt particle sizes [19].

**Figure 2:**
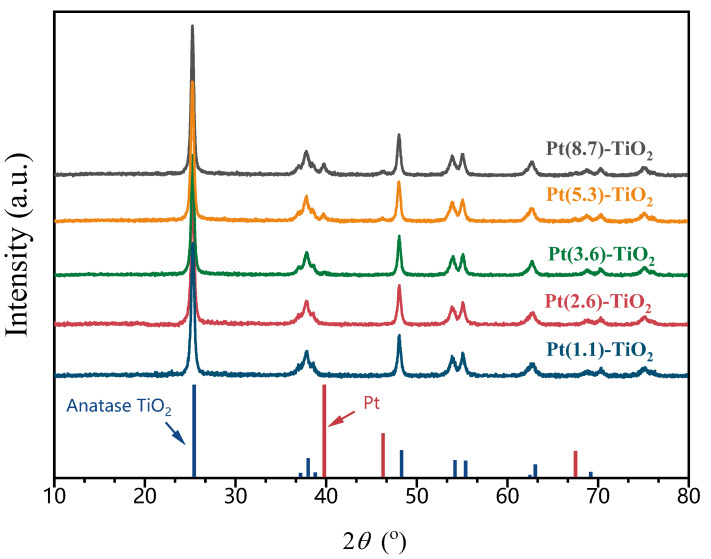
XRD patterns of Pt-TiO_2_ with different Pt particle sizes.

The XRD patterns of five catalysts with different Pt particle sizes, as shown in Figure 2, indicate the presence of six distinct diffraction peaks at 25.3°, 37.8°, 48°, 53.9°, 55°, and 62.7°. These peaks can be attributed to the characteristic diffraction peaks of anatase TiO_2_ (PDF#97-018-3767). Comparison of the XRD patterns of the five catalysts indicates that there were no significant alterations in the intensities of their characteristic peaks. This observation suggests that the catalyst preparation process carried out in different solvents for the hydrothermal/solvothermal process did not result in any structural damage to the TiO_2_. Furthermore, the XRD patterns of Pt-TiO_2_, synthesized using dilute ammonia and water as solvents, did not exhibit any distinctive diffraction peaks associated with Pt species. This finding implies that small-sized Pt particles are present on the surface of TiO_2_, which is consistent with the TEM results. Upon switching the solvent to methanol, an additional small broad peak emerges in the XRD pattern at 39.8°, aligning with the distinctive diffraction peak of Pt (111) [PDF#97-018-3075]. Furthermore, when the solvent is altered to ethanol, the intensity of the diffraction peak at 39.8° intensifies, accompanied by the appearance of a small broad peak at 46.3°. This outcome further substantiates the dispersion of Pt particles on the TiO_2_ surface, which is in accordance with the findings from the TEM images (Pt particle size of 5.3 nm). When the solvent is replaced with ethylene glycol, the diffraction peak of Pt exhibits a greater intensity, indicating an increase in particle size. This increase was further confirmed by the TEM characterization result, which measured the particle size to be 8.7 nm. Pt crystal sizes were also estimated from the broadening of Pt (111) and (200) diffraction peaks by using the Scherrer formula from the XRD patterns. The Pt particle sizes of catalysts prepared by the solvothermal method using methanol, ethanol, and ethylene glycol as solvents were calculated to be 3.9, 6.4, and 12.3 nm, respectively. This is larger than the Pt size obtained from the TEM images, which may be due to the XRD method being more sensitive to large particles. Nevertheless, the variation in Pt size obtained by XRD and TEM is consistent. For Pt-TiO_2_ catalysts prepared solvothermally using dilute ammonia as a solvent and hydrothermal methods, the absence of characteristic peaks of Pt makes it impossible to calculate the corresponding Pt sizes via the Scherrer formula.

**Figure 3:**
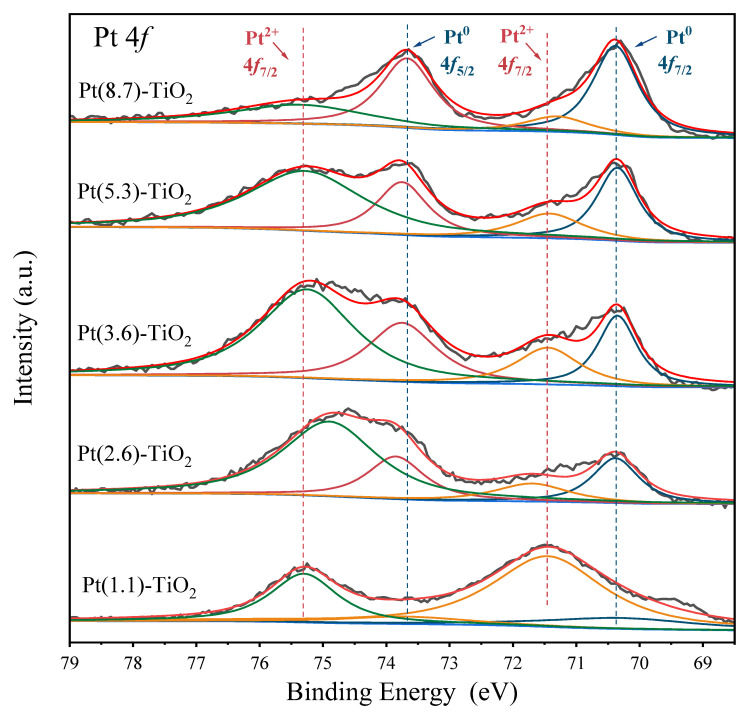
Pt 4*f* XPS spectra of Pt-TiO_2_ catalysts. Dark gray is the raw XPS spectra, red is the fitted XPS spectra, and light blue is the baseline selected for the XPS fit. Dark blue, dark red, orange, and green are the fitted peaks of Pt^0^ 4f_7/2_, Pt^0^ 4f_5/2_, Pt^2+^ 4f_7/2_, and Pt^2+^ 4f_5/2_, respectively.

The alteration in Pt particle size has a significant impact on its electronic structure. In Figure 3, the Pt 4*f* XPS spectra of the five catalysts display four distinct peaks at approximately 70.4, 73.8, 71.5, and 75.3 eV. These peaks correspond to the characteristic peaks of Pt^0^ 4*f*_7/2_, Pt^0^ 4*f*_5/2_, Pt^2+^ 4*f*_7/2_, and Pt^2+^ 4*f*_5/2_, respectively, in all catalysts [27,34]. The composition of Pt species varies significantly across the different sizes of Pt-TiO_2_ catalysts. In the Pt(1.1)-TiO_2_ catalyst, the Pt^2+^ species is predominant, with only a minor proportion of Pt^0^ (19%). However, as the Pt particle size increases from 2.6 nm to 3.6 nm, 5.3 nm, and 8.7 nm, the proportion of Pt^0^ gradually increases to 33%, 38%, 48%, and 69%, respectively (Table S2). Upon reaching an average particle size of 8.7 nm, it is noteworthy that Pt^0^ emerges as the predominant Pt species. This observation implies that smaller Pt nanoparticles demonstrate more pronounced interactions with the TiO_2_ surface, leading to a greater abundance of ionic Pt species. The interaction between Pt nanoparticles and the TiO_2_ surface intensifies with decreasing nanoparticle size, leading to a prevalence of ionic Pt. Conversely, larger particles demonstrate a diminished Pt-TiO_2_ interaction interface, resulting in a higher concentration of Pt^0^, in line with previous findings in the literature [34].

**Figure 4:**
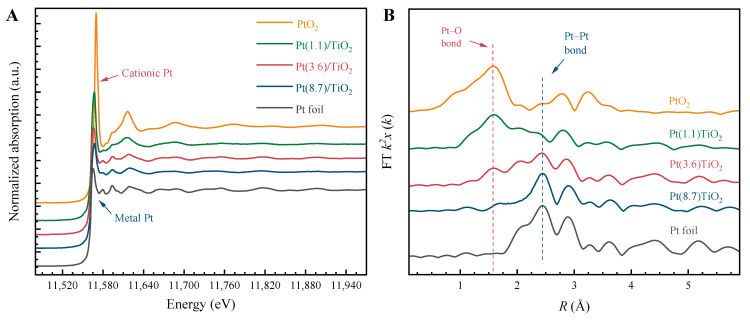
Normalized Pt-L_3_ edge XANES spectra (**A**), and Fourier transform magnitudes of the EXAFS spectra (**B**) of three Pd-TiO_2_ catalysts.

The X-ray absorption spectra (XAS) of three catalysts, namely Pt(1.1)-TiO_2_, Pt(3.6)-TiO_2_, and Pt(8.7)-TiO_2_, are depicted in Figure 4. Notably, their peak shapes and characteristic peak positions exhibit significant variations. Upon examining the X-ray near edge absorption spectra (XANES) presented in Figure 4A, it becomes evident that the peak shape of Pt species undergoes a gradual transition from PtO_2_ to Pt foil as the Pt particle size increases from 1.1 nm to 8.7 nm. Furthermore, the intensity of the white line peak gradually diminishes, while the peak position progressively shifts towards lower energy levels [35]. Furthermore, the extended-edge X-ray absorption fine-structure spectrum (EXAFS) of Pt in Pt(1.1)-TiO_2_ reveals that the Pt species predominantly exhibit coordination with O (Pt–O), with the Pt–Pt coordination peaks being relatively weak. This observation suggests that the Pt species are primarily in the ionic state. With the increase in the size of the Pt particles to 3.6 nm, the intensity of the Pt–O peak is significantly weakened, while the intensity of the Pt–Pt peak is significantly strengthened, which indicates a significant increase in the proportion of Pt^0^ in Pt particles. And the peak pattern of the spectrum in Pt(8.7)-TiO_2_ is close to that of Pt foil, which indicates that the Pt particles in this catalyst are mainly Pt^0^ species. The characterization findings obtained from XAS align with the outcomes from TEM and XRD.

### 2.2. Catalytic Performance of Alcohol Oxidation

**Figure 5:**
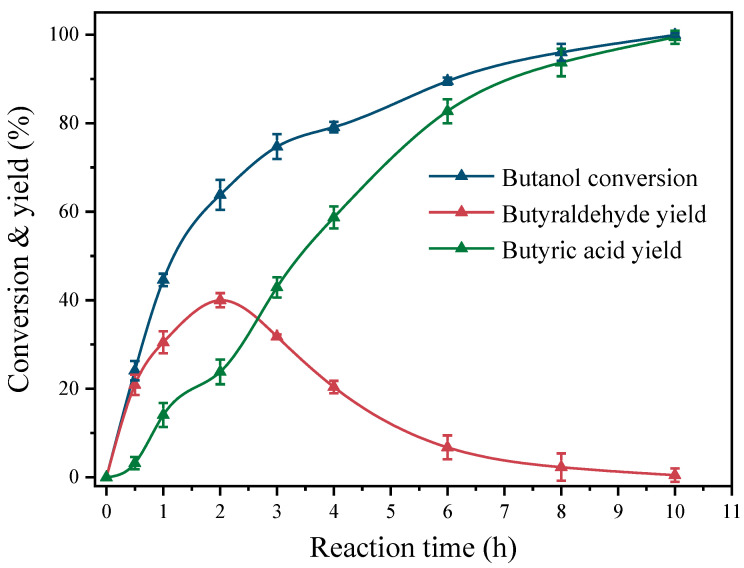
Oxidation performance of butanol catalyzed by Pt(3.6)-TiO_2_ with reaction time.

Table 1 displays the results of different Pt catalysts in the base-free catalysis of the butanol oxidation reaction in an aqueous environment. The catalytic activity of Pt-TiO_2_ is significantly affected by the size of the Pt particles. Specifically, the conversion of butanol on Pt(1.1)-TiO_2_ reached only 16.4% following a 10 h reaction at 80 °C. The selectivities of butyraldehyde and butyric acid were determined to be 92.3% and 7.7%, respectively (entry 1). Interestingly, under identical reaction conditions, the catalytic performance of butanol oxidation showed a trend of initially increasing and then decreasing with an increase in Pt particle size. The catalyst that demonstrated the highest performance in butanol oxidation among those investigated was the Pt(3.6)-TiO_2_ catalyst synthesized through the methanol solvothermal method. The catalyst demonstrated an exceptional butanol conversion of 99.5% and exhibited a high selectivity towards butyric acid at 99.3%. Moreover, the corresponding TOF reached 355 h^−1^ (entry 3), demonstrating the catalyst’s significant activity in the oxidation of butanol and its capacity to selectively generate butyric acid with a yield of 98.8%. Figure 5 depicts the temporal progression of butanol oxidation on the Pt(3.6)-TiO_2_ catalyst. The findings suggest that initially, butanol is oxidized to produce butyraldehyde, which then undergoes additional oxidation to generate butyric acid. Significantly, the highest yield of butyraldehyde, which amounted to 40%, was achieved after a reaction time of 2 h. Subsequently, a gradual decrease in the concentration of butyraldehyde occurred until it was fully oxidized to butyric acid. This observation is consistent with prior research on the oxidation mechanism of aliphatic alcohols [21]. The butanol conversions of Pt(2.6)-TiO_2_ and Pt(5.3)-TiO_2_, prepared through hydrothermal/solvothermal methods in water and ethanol, exhibited a decrease to 78.9% and 67.2%, respectively, in comparison to Pt(3.6)-TiO_2_. This decrease was accompanied by corresponding reductions in butyric acid selectivities and TOF values to 68.3% and 59.3%, and 281 and 165 h^−1^, respectively (entries 2 and 4). Furthermore, when the Pt particle size was further increased to 8.7 nm (ethylene glycol solvothermal method), the conversion of butanol and TOF was reduced to 42.8% and 123 h^−1^, respectively, compared to Pt(3.6)-TiO_2_ (entry 5), demonstrating relatively poor butanol oxidation activity.

Moreover, the impact of varying reaction temperatures on the oxidation efficiency of butanol in Pt(3.6)-TiO_2_ was investigated. Decreasing the reaction temperature from 80 to 60 and 40 °C resulted in an extended reaction time of 16 and 24 h, respectively, to achieve the near-complete conversion (>98%) of butanol. Additionally, the corresponding TOFs decreased to 135 and 54 h^−1^. Conversely, conducting the oxidation of butanol at 90 °C yielded TOFs that were 1.74 times higher compared to those at 80 °C, aligning with the principles of the Arrhenius formula. The reactivity of oxidation is significantly influenced by the temperature of the reaction, with an increase in temperature resulting in a corresponding increase in catalytic reactivity. It is worth mentioning that even at room temperature (30 °C), the catalyst demonstrated the ability to convert a large majority of butanol to butyric acid, achieving a yield of 98.4% after a duration of 40 h. This indicates its superior activity in the oxidation of non-activated alcohols. To the best of our knowledge, this catalyst is considered to be one of the most effective catalysts reported for the efficient catalytic oxidation of aliphatic alcohols at low temperatures. When air instead of O_2_ was used for the butanol oxidation, the conversion and TOF of butanol were reduced from 99.5% and 355 h^−1^ to 75.2% and 287 h^−1^, respectively (entry 10). Considering the safety and availability of air, it is more promising for the industrial butanol oxidation process. However, when the oxidant was changed to H_2_O_2_, the performance of butanol oxidation was significantly reduced with a conversion of only 3.1% after 10 h of reaction time. It is noteworthy that when the catalyst was added to the system, the H_2_O_2_ underwent a violent decomposition with a large number of bubbles generated. This may be due to the fact that the Pt catalyst rapidly catalyzes the decomposition of H_2_O_2_. This may be the reason why H_2_O_2_ and transition metal oxide systems are commonly used in the literature to catalyze the alcohol oxidation process. In addition, we synthesized four catalysts, namely Pt-SiO_2_, Pt-CeO_2_, Pt-ZrO_2_, and Pt-Al_2_O_3_, using the same preparation method employed for Pt(3.6)-TiO_2_. The evaluation results revealed that the TOFs for butanol oxidation after a 10 h reaction at 80 °C were 125, 328, 296, and 143 h^−1^, respectively (entries 12–15). This suggests that the catalytic oxidation activity of Pt is also influenced by the characteristics of supports. The examination of the catalytic activities of various supports indicates that the reducible supports, namely TiO_2_, CeO_2_, and ZrO_2_, exhibit superior performance compared to the non-reducible carriers (SiO_2_ and Al_2_O_3_). This disparity can be attributed to the synergistic interaction between Pt and the reducible supports [10].

### 2.3. Pt Size Effect on Catalytic Performance of Butanol

**Figure 6:**
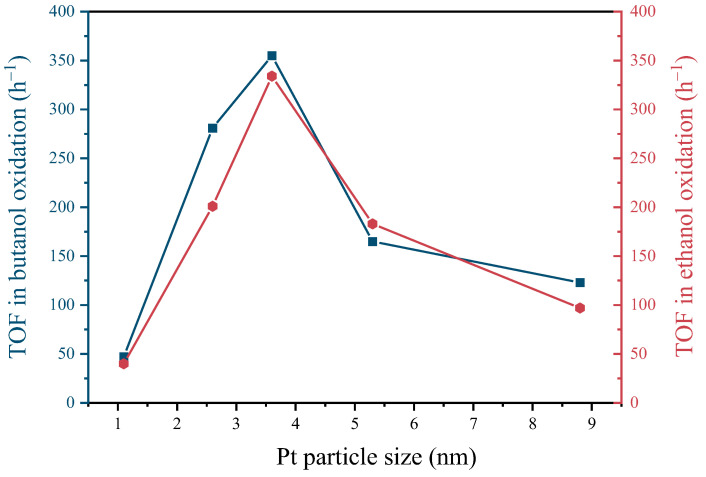
Plots of conversion of butanol and ethanol with Pt particle size in Pt-TiO_2_ catalysts.

The impact of Pt particle size on the catalytic efficacy of butanol and ethanol oxidation is examined in Figure 6. It can be observed that the TOF for the oxidation of both butanol and ethanol follows a pattern of an initial increase followed by a decrease, with the highest TOF observed at a Pt particle size of 3.6 nm. Pt(3.6)-TiO_2_ exhibited a notable enhancement in TOF for butanol oxidation, with approximately 7.6- and 2.9-times improvements compared to Pt(1.1)-TiO_2_ and Pt(8.7)-TiO_2_, respectively. Similarly, for ethanol oxidation, Pt(3.6)-TiO_2_ demonstrated an 8.4- and 3.5-fold increase in TOF compared to Pt(1.1)-TiO_2_ and Pt(8.7)-TiO_2_. This observed trend aligns with previous findings regarding the catalytic activity of Pt particles, suggesting that an optimal strength of interaction between Pt and the reactants is necessary for optimal catalytic performance, as interactions that are either too strong or too weak can lead to a decline in catalytic performance [34].

### 2.4. Catalytic Performance of Other Non-Activated Alcohols and Biomass-Based Alcohols

Furthermore, the oxidation of various aliphatic alcohols, including ethanol, propanol, pentanol, hexanol, and octanol, was assessed over Pt(3.6)-TiO_2_ (Table 2). The conversions of these alcohols ranged from 91.6% to 95.8% with TOFs of more than 310 h^−1^, while the corresponding yields of organic acids reached values between 83.5% and 94.3% at a temperature of 80 °C for 10 h (entries 1–6). These results suggest that Pt(3.6)-TiO_2_ exhibits exceptional oxidative activity towards commonly non-activated aliphatic primary alcohols. Additionally, the catalytic performance of Pt(3.6)-TiO_2_ in the oxidation of 2-octanol and cyclohexanol demonstrated that the corresponding oxidation products for secondary alcohols were ketones, with yields exceeding 99% for both substrates (entries 7–8). It is noteworthy to mention that the TOFs of the aforementioned secondary alcohols are 14–31% greater than those of primary alcohols, aligning with previous literature findings that suggest secondary alcohols exhibit relatively easier activation [11]. In addition, the oxidation performance of other biomass-based alcohols catalyzed by Pt(3.6)-TiO_2_ was also investigated. For furfural, phenol, 5-hydroxymethylfurfural, furfuryl alcohol, and tetrahydrofurfuryl alcohol, the conversions were in the range of 81.2–99.6% with TOFs between 314 and 435 h^−1^ for a reaction of 10 h at 60 °C. For polyhydroxy biomass-based alcohols, such as glycerol, the oxidation products are more complex. The glycerol conversion was 62% after 10 h of reaction time at 80 °C. The resulting product contained glyceric acid, lactic acid, glycolic acid, acetic acid, and formic acid with selectivities of 64.2%, 2.8%, 27.4%, 1.5%, and 4.1%, respectively. The oxidation of glucose was carried out at 60 °C, and after 10 h of reaction time, a glucose conversion of 64.5% and a gluconic acid selectivity of 71.9% could be achieved over Pt(3.6)-TiO_2_. Continued prolongation of the reaction time or an increase in the reaction temperature resulted in a substantial decrease in gluconic acid selectivity and the generation of a number of carbon–carbon bond breaking products.

### 2.5. Reusability and Stability of Pt(3.6)-TiO_2_

**Figure 7:**
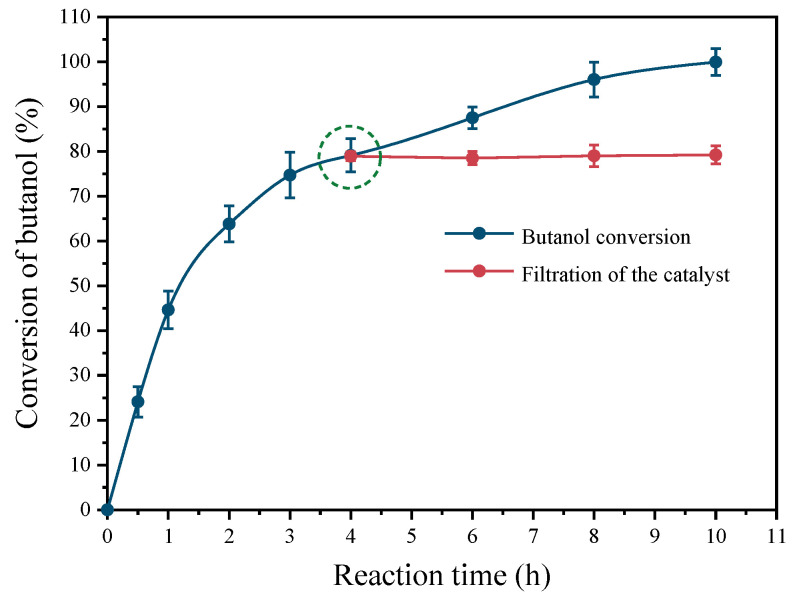
Performance of leaching of Pt(3.6)-TiO_2_ after removal from reactor at reaction time of 4 h. The dotted circle represents removing the catalyst from the reaction system.

Figure 7 depicts the leaching experiment conducted over the Pt(3.6)-TiO_2_ catalyst. The conversion of butanol oxidation reached 79.1% after 4 h of reaction time. If the catalyst powder were to be removed from the reactor at this point and the reaction were continued by charging with 0.5 MPa O_2_, the butanol conversion would remain within the range of 78.9–79.2% after another 6 h of reaction time. In order to ensure the reliability of the obtained data, the evaluation was repeated three times under identical conditions. The Pt loading of the used Pt(3.6)-TiO_2_ after 4 h of reaction time was still 0.95 wt%, indicating that there was no significant leaching of Pt (Table S1, entry 10). In addition, the detection of residual Pt in the supernatant after the reaction revealed that the concentration of Pt was not detected. These results demonstrated that there was no significant leaching of Pt species into the reaction solution.

**Figure 8:**
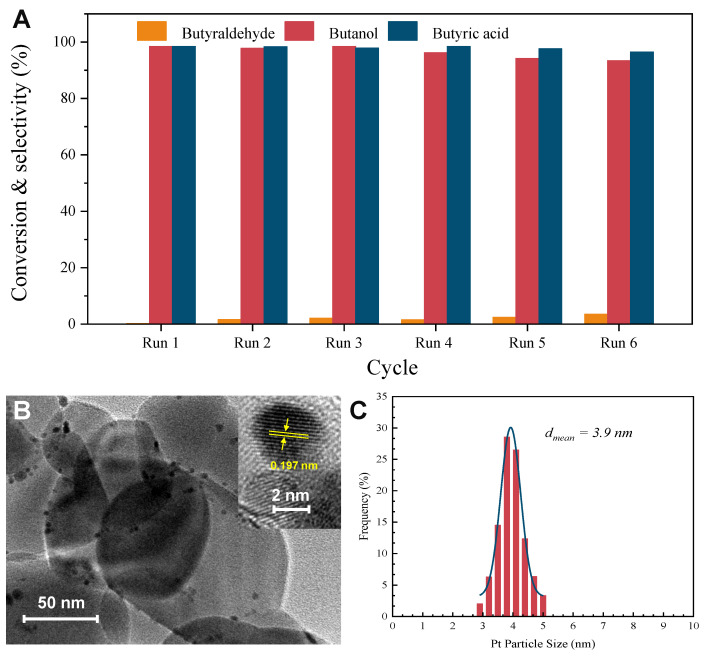
Stability of Pt(3.6)-TiO_2_ for aerobic oxidation of butanol (**A**). Reaction conditions: 50 mg of Pt-TiO_2_ catalysts, certain amount of aliphatic alcohol, 8 mL of H_2_O, and 0.5 MPa of O_2_. After reaction, the catalyst was centrifuged, washed several times with deionized water and ethanol, then dried in an oven at 100 °C. For the second reaction, catalyst, substrate, and internal standard were reintroduced into the reactor, which was repeated six times in this order. TEM image of Pt(3.6)-TiO_2_ after six cycle reactions (**B**); the inset is the high-resolution TEM image. Pt particle size of used Pt(3.6)-TiO_2_ is shown in (**C**).

Figure 8A illustrates that the catalyst demonstrated remarkable catalytic stability after six cycles, with only a minor decline in activity and a decrease in butyric acid selectivity. Specifically, the conversion of butanol and the selectivity of butyric acid slightly decreased from 99.5% and 99.3% to 93.4% and 96.5%, respectively. The stability of the Pt(3.6)-TiO_2_ catalyst was confirmed by the HR-TEM results (Figure 8B), which showed that the Pt particles remained highly dispersed on the TiO_2_ support even after six cycles. Additionally, the average size of the Pt particles only slightly changed from 3.6 nm to 3.9 nm for the fresh catalysts, indicating minimal agglomeration (Figure 8C). Based on the findings from the leaching and stability experiments, it can be concluded that the Pt(3.6)-TiO_2_ catalyst prepared in this study exhibits efficient and stable catalytic activity for the oxidation of aliphatic alcohols at low temperatures, which shows a notable propensity for converting primary alcohols into aliphatic acids with remarkable selectivity.

Table 3 presents a comparison between the catalytic performance of the prepared Pt(3.6)-TiO_2_ and previously reported non-activated aliphatic alcohol oxidation catalysts. The results indicate that the alcohol oxidation reaction was conducted under varying conditions, such as different reaction temperatures, molar ratios of alcohols to metal active centers, the presence or absence of a base, and reaction times. These diverse conditions have the potential to greatly influence the catalytic performance of alcohol oxidation. At low temperatures (<60 °C), catalysts with high activity usually require the addition of a base or alkaline treatment of the catalyst. Pt(3.6)-TiO_2_ catalysts can efficiently convert butanol to butyric acid without adding a base, even at room temperature. This demonstrates the good low-temperature oxidation activity of Pt(3.6)-TiO_2_. Furthermore, among the aliphatic oxidation catalysts, Pt-based catalysts have the highest percentage reported in the literature, which may be related to the high oxidation potential of Pt [36]. Comparing the reported Pt-based catalysts, including monometallic Pt and Pt-based bimetallic catalysts, Pt(3.6)-TiO_2_ is highly competitive. Such a comprehensive comparison clearly demonstrates that the Pt(3.6)-TiO_2_ catalyst prepared by the one-step methanol solvothermal method has one of the best oxidation performances among all currently reported non-activated alcohol oxidation catalysts, particularly its low-temperature performance, high alcohol-to-metal molar ratio, alcohol conversion, and organic acid selectivity.

**Figure 9:**
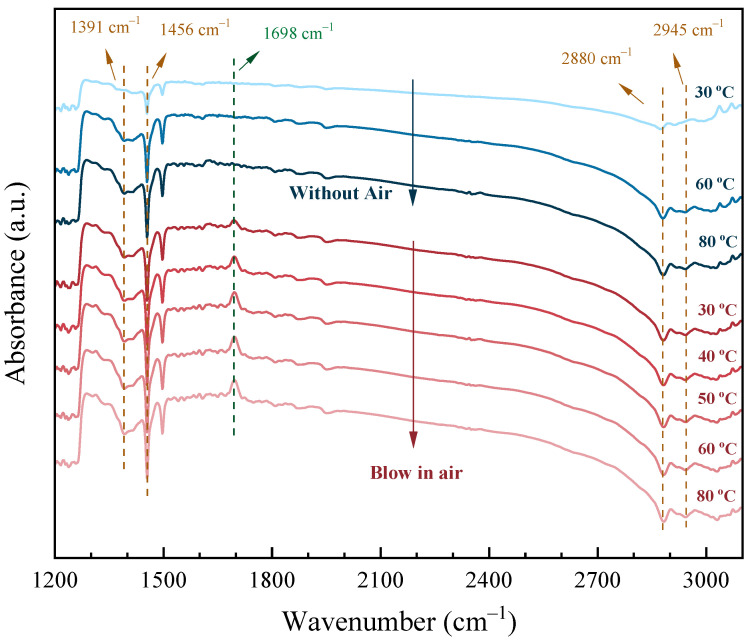
IR spectra of ethanol adsorption over Pt(3.6)-TiO_2_ at different temperatures within or without air. Reaction conditions: The Pt(3.6)/TiO_2_ catalyst was first purged with Ar gas (30 mL min^−1^) at 300 °C to remove adsorbents on the surface. Ethanol vapor (25 °C) was introduced into the in situ reaction cell by means of Ar gas bubbling, and air was introduced into the sample surface through a gas injector.

To further illustrate the excellent performance of Pt(3.6)-TiO_2_ in aliphatic alcohol oxidation, in situ infrared spectroscopy was employed to observe the adsorption and reaction process of ethanol on the catalyst surface. As shown in Figure 9, the vibrational peaks with spectral peaks located at 1391–1456 cm^−1^ and 2880–2945 cm^−1^ are attributed to the characteristic vibrational peaks of saturated C–C and C–H bonds, respectively, while the peaks at 1698 cm^−1^ are attributed to the characteristic vibrational peaks of the aliphatic carbonyl group [41,42]. When ethanol was adsorbed on the surface of the catalyst alone, the IR spectra did not change significantly as the IR reaction cell temperature increased from 30 to 80 °C (blue region). When ethanol was adsorbed on the surface of the catalyst and then air was injected into the infrared reaction cell (red region), a vibrational peak appeared in the spectrum at 1698 cm^−1^ quickly, which indicated that the ethanol was oxidized to acetaldehyde at this time. As the temperature gradually increased to 80 °C, the carbonyl vibrational peak was subsequently enhanced, indicating that more acetaldehyde was generated. This indicates that more acetaldehyde was generated on the surface of Pt(3.6)-TiO_2_. In contrast, no obvious carbonyl vibrational peaks were found for Pt(1.1)-TiO_2_ and Pt(8.7)-TiO_2_ after ethanol adsorption at 30 °C with the passage of air (Figure S2), which further illustrates the excellent low-temperature oxidation performance of Pt(3.6)-TiO_2_ for aliphatic alcohols.

### 2.6. Discussion of the Catalytic Mechanism of Pt(3.6)-TiO_2_

**Figure 10:**
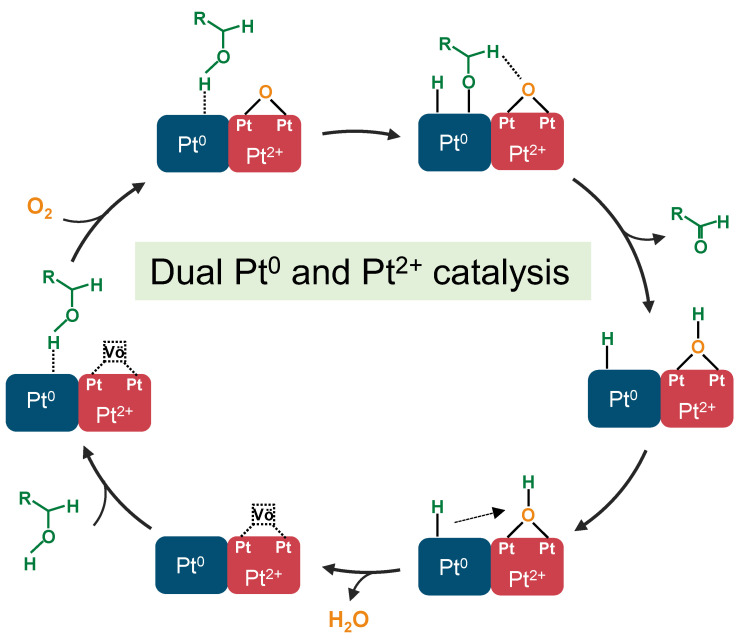
Schematic diagrams of catalytic mechanism over dual Pt^0^ and Pt^2+^ sites of Pt(3.6)-TiO_2_. Vö represents the oxygen vacancies on PdO.

In the study of alcohol oxidation catalysts, the influence of the electronic structure of metal species on the catalytic activity and the role they play in the catalytic process are still a topic of discussion. Some scholars believe that Pt^0^ or Pd^0^ is the active site for alcohol oxidation, and its oxidation mechanism mainly involves the adsorption and activation of hydroxyl hydrogen by M^0^, first forming alkoxyl species and hydrogen species, followed by the further elimination of β-H species and the formation of aldehydes; the adsorption of hydrogen species is oxidized to water by oxygen, which releases the surface of M^0^ and completes the catalytic cycle [18]. Other scholars have suggested that the ionic metal first activates molecular oxygen into reactive oxygen species; the reactive oxygen species removes hydroxy hydrogen and β-H, respectively, to form aldehydes and water [43,44]. However, the results of the present study do not seem to conform to the two typical mechanisms mentioned above. From the catalytic activity results for butanol oxidation, it can be seen that Pt(3.6)-TiO_2_, with 38% Pt^0^ and 62% Pt^2+^, exhibits the best catalytic activity and the highest TOF. Continuing to increase the percentage of either Pt^0^ or Pt^2+^ leads to poorer catalytic performance. This is consistent with recent results reported in the literature that the presence of both Pd^0^ and Pd^2+^ significantly enhances the alcohol oxidation activity [45,46]. Those two studies proposed a mechanism for the synergistic catalysis of alcohol oxidation by Pd^0^ and Pd^2+^. In addition, there are also some studies from the literature demonstrating that the presence of ionic state metals can significantly promote the β-H elimination process. Combining the synergistic catalytic mechanism of Pd^0^ and Pd^2+^ proposed in the literature and the conformational relationship between the butanol oxidation activity and the electronic structure of the catalyst in this study, we believe that a two-catalytic site mechanism is more reasonable in the Pt-TiO_2_ system. Its catalytic cycle mechanism is as follows (Figure 10): the hydroxyl hydrogen of butanol is first adsorbed on Pt^0^ to form hydrogen and butyl oxygen, the molecular oxygen is activated on the oxygen vacancies (Vö) of PtO to generate active lattice oxygen, the lattice oxygen promotes the elimination of β-H to generate butyraldehyde and hydrogen, and the two hydrogens and one lattice oxygen generate water to complete the catalytic cycle.

## 3. Materials and Methods

All chemical reagents (analytically pure) and catalyst supports, including nano TiO_2_ (99.8% metal basis, 60 nm, anatase, hydrophilic), SiO_2_ (fumed silica, hydrophilic, specific surface area: 400 m^2^ g^−1^), Al_2_O_3_ (nano alumina, 99.99% metal basis, γ phase, 40 nm), CeO_2_ (cerium oxide, 99.9%, 50 nm), and CeO_2_ (zirconium dioxide nanoparticles, 99.9%, 20 nm, monoclinic phase), were purchased from McLean Reagents Ltd. All samples were used directly without further treatment.

### 3.1. Preparation of the Catalyst

**Figure 11:**
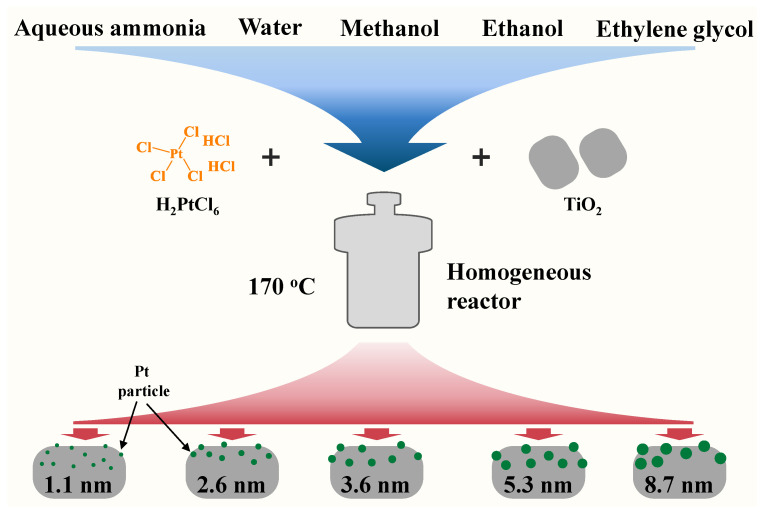
Schematic diagram of hydrothermal/solvothermal synthesis of Pt-TiO_2_ catalysts with different sizes.

Catalysts with different Pt particle sizes were prepared using a simple one-step hydrothermal/solvothermal method as previously reported in the literature with some modifications [47]. For example, Pt(3.6)-TiO_2_ was prepared as follows: 1 g of TiO_2_ nanoparticles was dispersed in anhydrous methanol, and a certain amount of aqueous H_2_PtCl_6_ was added under stirring, after which the dispersion was transferred to a stainless-steel reactor containing a polytetrafluoroethylene liner. The reactor was placed within a homogeneous reactor (rotating oven) and subjected to solvothermal rotation at a temperature of 170 °C and a speed of 15 r/min for 24 h. Subsequently, the reactor was gradually cooled to room temperature, and the resulting samples were subjected to centrifugation, washing, and subsequent drying at a temperature of 100 °C for 12 h. It should be noted that the synthesis reactor must be allowed to cool completely before proceeding. The preparation of Pt-TiO_2_ catalysts with average Pt particle sizes of 1.1, 2.6, 5.3, and 8.7 nm followed a similar procedure to that of the 3.6 nm catalyst, with the exception of the solvents employed in the synthesis process. The Pt catalysts with corresponding size were obtained by replacing methanol with ammonia (5% concentrated ammonia, v/v), water, ethanol, and ethylene glycol, respectively. A schematic diagram of the preparation process of Pt-TiO_2_ with different Pt sizes is shown in Figure 11. The preparation of different support-loaded Pt catalysts was similar to that of Pt(3.6)-TiO_2_, which only required a change of the TiO_2_ nanoparticles to the corresponding other supports.

### 3.2. Characterization Techniques

Transmission electron microscopy (TEM) characterization of the samples was characterized on a JEM-F200. X-ray diffraction peaks were obtained from a Bruker D8 ADVANCE XRD instrument with a tube voltage of 40 kV, 2 *θ* angles of 5–90°, and a scan rate of 5°/min. X-ray photoelectron spectroscopy characterization was performed on a Thermo Fisher Scientific K-Alpha. Inductively coupled plasma (AtomScan-16) was used to determine the actual loading of Pt in the catalysts. The actual loadings of Pt for Pt(1.1)-TiO_2_, Pt(2.6)-TiO_2_, Pt(3.6)-TiO_2_, Pt(5.3)-TiO_2_, Pt(8.7)-TiO_2_, and used Pt(3.6)-TiO_2_ are 0.41, 0.83, 0.95, 0.92, 0.97, and 0.92 wt%, respectively. X-ray absorption spectra were obtained at the BL14W1 line station of the Shanghai Light Source using a monochromator with a Si (111) crystal face with an energy resolution higher than 2 × 10^−4^. The absorption peaks of the Pt elements were acquired in fluorescence mode. In situ IR spectroscopy was performed on a Brooks Vertex 70 IR spectrometer using ethanol as a probe molecule. The Pt(3.6)/TiO_2_ catalyst was first purged with Ar gas (30 mL min^−1^) at 300 °C to remove adsorbents on the surface. Ethanol vapor (25 °C) was introduced into the in situ reaction cell by means of Ar gas bubbling, and air was introduced into the sample surface through a gas injector to investigate the mechanism of ethanol oxidation.

### 3.3. Catalytic Reaction

An amount of 50 mg of catalyst was added to a 30 mL pressurized reactor with 8 mL of deionized water, a quantity of aliphatic alcohol, and p-xylene (internal standard). The reactor was tightened and filled with 0.5 MPa of O_2_. After that, it was then placed on a heating module with thermocouples to set the temperature (30–90 °C) for the reaction. At the end of the reaction, the reactor was cooled to room temperature in an ice water bath. In order to increase the solubility of the aliphatic alcohols in water, an amount of 1,4-dioxane was added to the suspension, which was centrifuged to obtain the supernatant, and the sample was analyzed using a Haixin gas chromatograph (GC-950) fitted with a DB-1 capillary column and a hydrogen flame ion detector. The GC calibration curves of butanol oxidation are shown in Figure S3. For the 5-hydroxymethylfurfural, glycerol, and glucose oxidation reaction, the products were analyzed by high-performance liquid chromatography. The analytical method referred to the method reported by Yuan and co-workers [48]. When the alcohol oxidation reaction was finished, the catalyst was centrifuged, washed several times with deionized water and ethanol, then dried in an oven at 100 °C. The used catalyst, substrate, and internal standard were reintroduced into the reactor for the second reaction, which was repeated six times in this order.

The conversion (*C*) of alcohol, the selectivity (*S*) and yield (*Y*) of organic acid, as well as the turnover frequency (*TOF*) were calculated as follows [37]:

(1) $$C=\frac{n_{0,S}-n_{t,S}}{n_{0,S}}=\left(1-\frac{F_{s}A_{s}}{F_{s}A_{s}+\sum F_{i}A_{i}}\right)\times 100\%$$

(2) $$S=\frac{n_{P_{1}}}{n_{P_{1}}+\sum n_{P_{i}}}=\left(\frac{F_{P_{1}}A_{P_{1}}}{F_{P_{1}}A_{P_{1}}+\sum F_{P_{i}}A_{P_{i}}}\right)\times 100\%$$

(3) Y=C×S×100%

(4) $$TOF=\frac{n_{0,S}-n_{t,S}}{t\times n_{\mathrm{Pt}}}$$

The variables are defined as follows: *n* represents the quantity of substrate or product moles, *n*_0_ represents the initial quantity of moles, t represents the duration of the reaction, *F* represents the relative correction factor, *A* represents the peak area, *S* represents the substrate, *P* represents the product, *P*_1_ represents the organic acid, *P_i_* represents the by-product, and *n*_Pt_ represents the quantity of moles of Pt present in the catalyst.

## 4. Conclusions

In summary, we successfully synthesized Pt-TiO_2_ catalysts through a one-step hydrothermal/solvothermal method, which exhibited remarkable catalytic oxidation efficiency towards aliphatic alcohols derived from biomass. By altering the solvent used in the hydrothermal/solvothermal process, we were able to effectively control the size of Pt particles, ranging from 1.1 nm to 8.7 nm. Among the catalysts studied, the Pt(3.6)-TiO_2_ catalyst, consisting of Pt particles with a diameter of 3.6 nm and prepared through the methanol solvothermal method, exhibited excellent catalytic activity for the oxidation of butanol. Even at room temperature, the Pt(3.6)-TiO_2_ catalyst maintained a high butanol conversion rate of 98.9% and a butyric acid selectivity of 98.4%. The high activity of Pt(3.6)-TiO_2_ can be attributed to the oxidation state of Pt, which facilitates synergistic catalysis between Pt^0^ and Pt^2+^.

## Figures and Tables

**Table 1:** Performance of various Pt-TiO_2_ in the oxidation of butanol.

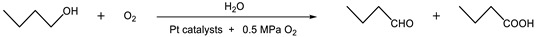									
Entry	Catalyst	Butanol/Pt	Temperature (°C)	Time (h)	Conversion (%)	Selectivity (%)		Yield (%) Butyric Acid	TOF ^3^ (h^−1^)
						Butyraldehyde	Butyric Acid		
1	Pt(1.1)-TiO_2_	500	80	10	16.4	92.3	7.7	1.3	47
2	Pt(2.6)-TiO_2_	500	80	10	78.9	31.7	68.3	53.9	281
3	Pt(3.6)-TiO_2_	500	80	10	99.5	0.7	99.3	98.8	355
4	Pt(5.3)-TiO_2_	500	80	10	67.2	40.7	59.3	39.8	165
5	Pt(8.7)-TiO_2_	500	80	10	42.8	57.3	42.7	18.3	123
6	Pt(3.6)-TiO_2_	50	30	40	98.9	1.6	98.4	97.3	36
7	Pt(3.6)-TiO_2_	100	40	24	99.8	0.9	99.1	98.9	54
8	Pt(3.6)-TiO_2_	200	60	16	98.7	0.5	99.5	98.2	135
9	Pt(3.6)-TiO_2_	1000	90	10	99.0	1.2	98.8	97.8	619
10	Pt(3.6)-TiO_2_ ^1^	500	80	10	75.2	42.1	57.9	43.5	287
11	Pt(3.6)-TiO_2_ ^2^	500	80	10	3.1	99.5	–	–	–
12	Pt-SiO_2_	500	80	10	41.6	37.4	62.6	32.9	125
13	Pt-CeO_2_	500	80	10	90.7	13.8	86.2	74.7	328
14	Pt-ZrO_2_	500	80	10	88.1	15.9	84.1	69.0	296
15	Pt-Al_2_O_3_	500	80	10	58.4	28.8	71.2	48.7	143

Reaction conditions: 50 mg of Pt-TiO_2_ catalysts, certain amount of butanol, 8 mL of H_2_O, and 0.5 MPa of O_2_. The actual loadings of Pt for entries 1–5 are 0.41, 0.83, 0.95, 0.92, and 0.97 wt%, respectively, while the actual loadings of Pt for entries 12–15 are 0.90, 0.98, 0.96, and 0.94 wt%, respectively. ^1^ 0.5 MPa of air. ^2^ 50 mg of Pt-TiO_2_ catalysts, certain amount of butanol, 8 mL of a 0.0075% aqueous H_2_O_2_ solution with a molar ratio of H_2_O_2_/butanol of 5. ^3^ TOF calculated by initial reaction of 30 min.

**Table 2:** The catalytic oxidation performance of different alcohols on Pt(3.6)-TiO_2_.

Entry	Substrate	Conversion (%)	Selectivity (%)		Yield (%) Acid	TOF (h^−1^) ^1^
			Aldehyde/Ketone	Acid		
1	Ethanol	92.5	5.6	94.4	87.3	334
2	Propanol	91.6	6.4	93.6	85.7	341
3	Butanol	99.5	0.7	99.3	98.8	355
4	Pentanol	92.1	9.3	90.7	83.5	320
5	Hexanol	93.4	3.7	96.3	89.9	317
6	Octanol	95.8	1.6	98.4	94.3	346
7	2-Octanol	99.7	99.9 ^2^	–	99.6	416
8	Cyclohexanol	99.3	99.9 ^2^	–	99.2	403
9 ^3^	Furfural	99.6	–	99.3	98.9	435
10 ^4^	Phenol	81.2	76.4	–	62.0	314
11 ^4^	5-Hydroxymethylfurfural	96.4	–	99.4 ^5^	95.8	346
12 ^4^	Furfuryl alcohol	99.2	1.3	98.7	97.9	394
13 ^4^	Tetrahydrofurfuryl alcohol	90.3	7.6	92.4	83.4	327
14 ^6^	Glycerol	62.0	–	99.8	61.9	179
15 ^7^	Glucose	64.5	–	71.9	46.4	162

Reaction conditions: 50 mg of Pt-TiO_2_ catalysts, certain amount of alcohol, the molar ratio of substrate/Pt of 500, 8 mL of H_2_O, 80 °C, 10 h, and 0.5 MPa of O_2_. ^1^ TOF calculated by initial reaction of 30 min. ^2^ selectivity refers to the corresponding ketone. ^3^ the molar ratio of furfural/Pt of 1000 and 60 °C. ^4^ the reaction temperature was 60 °C. ^5^ the products contain 2,5-furandicarboxylic acid and 5-formyl-2-furancarboxylic acid with a ratio of 8.6: 1. ^6^ the products contain glyceric acid, lactic acid, glycolic acid, acetic acid, and formic acid with selectivities of 64.2%, 2.8%, 27.4%, 1.5%, and 4.1%, respectively. ^7^ the molar ratio of glucose/Pt of 300 and 60 °C.

**Table 3:** Comparison of catalytic performance for the aerobic oxidation of aliphatic alcohol over various catalysts.

Entry	Catalyst	Temp. (°C)	Molar Ratio	Base	Time (h)	Conv. (%)	Sel. (%)	TOF (h^−1^)	Ref.
1	Pt/Bi_2_O_3_	90	75	free	5	99.0	99.0	–	[21]
2	Au-Pd/TiO_2_	100	550	free	6	89.7	92.5	250	[1]
3	Pt sol	80	20	free	24	100	99.7	37	[22]
4	Au/SBA-15-Py	130	2190	NaAc ^4^	24	50.7	98.0	–	[25]
5	Pt-Pd/TiO_2_	100	163	free	6	75	53	–	[3]
6 ^1^	Pd/CK05-550	160	200	free	5	96	71	–	[24]
7 ^1^	(PtCu)^L^@C^K^	140	14,600	free	17	40.9	7.3	–	[23]
8 ^1^	Ru/Mg_1−*x*_Fe*_x_*O	150	400	free	4	94.1	95.6	–	[37]
9 ^2^	AuPd@HT-PO_4_^3−^	55	10	free	24	62	42	–	[20]
10 ^2^	Au/NiO	100	1000	free	18	90	68	–	[38]
11 ^2^	PdBiTe/C	60	100	K_2_CO_3_	8	>90	90	–	[39]
12 ^3^	Pt^(NP)^@PMO-IL-2	90	20	K_2_CO_3_	21	83	>99 ^5^	–	[40]
13	Pt(3.6)/TiO_2_	30	50	free	40	98.9	98.4	36	This work
14	Pt(3.6)/TiO_2_	80	500	free	10	99.5	99.3	355	
15	Pt(3.6)/TiO_2_	90	1000	free	10	99	98.8	619	

^1^ entries 6–8 refer to the oxidation of ethanol. ^2^ entries 9–11 refer to the oxidation of *n*-octanol. ^3^ entry 12 refers to the oxidation of 2-octanol. ^4^ NaAc represents sodium acetate. ^5^ this selectivity refers to 2-octanone.

## Data Availability

The data presented in this study are available in the Supplementary Materials.

## Supplementary Materials

The following supporting information can be downloaded at: https://www.mdpi.com/article/10.3390/molecules29071450/s1, Table S1: ICP-OES results of Pt content for Pt-TiO_2_ catalysts and supernatant; Table S2: Results of Pt 4*f* XPS spectra of Pt-TiO_2_ catalysts; Figure S1: Pt particle size of series of Pd-TiO_2_: (A) Pt(1.1)-TiO_2_; (B) Pt(2.6)-TiO_2_; (C) Pt(3.6)-TiO_2_; (D) Pt(5.3)-TiO_2_; (E) Pt(8.7)-TiO_2_; Figure S2: IR spectra of ethanol adsorption over Pt(1.1)-TiO_2_ and Pt(8.7)-TiO_2_ at 30 °C within air; Figure S3: The GC calibration curves of butyraldehyde, butanol, butyric acid, and butyl butyrate.
