# Supplementary material for: One-Step Hydrothermal/Solvothermal Preparation of Pt/TiO2: An Efficient Catalyst for Biobutanol Oxidation at Room Temperature
Source: Molecules. 2024 Mar 24;29(7):1450. doi: 10.3390/molecules29071450 (PMC11013154; doi:10.3390/molecules29071450)
Supplement: Supplementary file 1 [file molecules-29-01450-s001.zip › molecules-2870474-supplementary.pdf]

Supplementary Materials

One-step hydrothermal/solvothermal preparation of Pt/TiO_2: an efficient catalyst for bio-butanol oxidation at room temperature

Lijun Lei [1,*], Qianyue Cao [2] and Jiachen Ma [1], Fengxiao Hou [1]

[1] School of Energy and Power Engineering, North University of China, Taiyuan 030051, China; leilijun@nuc.edu.cn (L.L.); neromjc@gmail.com (J.M.); mercatushou@outlook.com (F.H.)

[2] School of Chemistry and Chemical Engineering, North University of China, Taiyuan 030051, China; Caoqianyuedw@163.com (Q.C.)

* Correspondence: leilijun@nuc.edu.cn (L.L.)

Table S1. ICP-OES results of Pt content for Pt-TiO_2 catalysts and supernatant.

Entry	Catalyst	Theoretical loading (wt.%)	Actual loading (wt.%)	Pt concentration (mg/L) [2]
1	Pt(1.1)-TiO_2	1.0	0.41	–
2	Pt(2.6)-TiO_2	1.0	0.83	–
3	Pt(3.6)-TiO_2	1.0	0.95	–
4	Pt(5.3)-TiO_2	1.0	0.92	–
5	Pt(8.7)-TiO_2	1.0	0.97	–
6	Pt-SiO_2	1.0	0.90	–
7	Pt-CeO_2	1.0	0.98	–
8	Pt-ZrO_2	1.0	0.96	–
9	Pt-Al_2O_3	1.0	0.94	–
10 [1]	Pt(3.6)-TiO_2	1.0	0.95	–
11 [2]	Pt(3.6)-TiO_2	–	0.92	–
12	Supernatant	–	–	Not detected

[1] After 4 hours of butanol oxidation.

[2] After six cycles of butanol oxidation.

[2] Before conducting ICP-OES testing, the supernatant was diluted to 100 mL.

Table S2. Results of Pt 4*f* XPS spectra of Pt-TiO_2 catalysts.

Entry	Catalyst	Pt^0 peak position		Pt^{2+} peak position		Pt^0 fraction (%)
		$4f_{7/2}$	$4f_{5/2}$	$4f_{7/2}$	$4f_{5/2}$	
1	Pt(1.1)-TiO_2	70.3	73.7	71.5	75.3	19
2	Pt(2.6)-TiO_2	70.4	73.8	71.7	75.0	33
3	Pt(3.6)-TiO_2	70.4	73.8	71.5	75.3	38
4	Pt(5.3)-TiO_2	70.4	73.8	71.5	75.3	48
5	Pt(8.7)-TiO_2	70.4	73.7	71.4	75.3	69

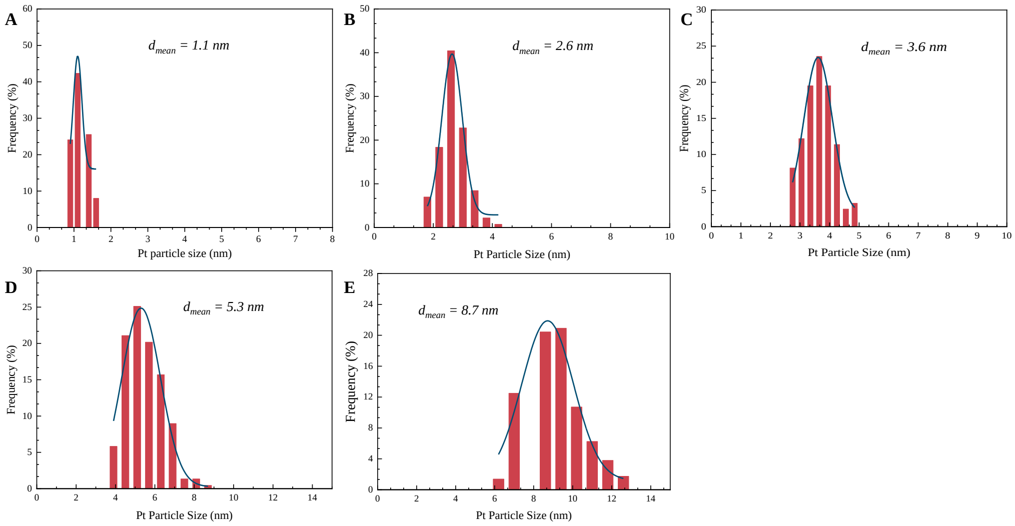

Figure S1. Pt particle size of series of Pd-TiO_2, (A) Pt(1.1)-TiO_2; (B) Pt(2.6)-TiO_2; (C) Pt(3.6)-TiO_2; (D) Pt(5.3)-TiO_2; (E) Pt(8.7)-TiO_2; (F) used Pt(3.6)-TiO_2.

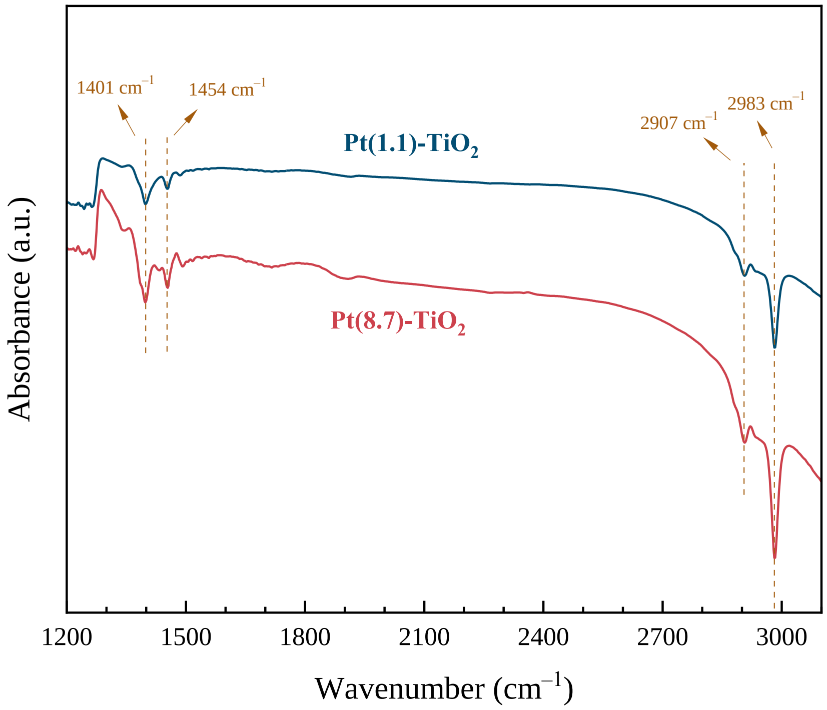

Figure S2. IR spectra of ethanol adsorption over Pt(1.1)-TiO_2 and Pt(8.7)-TiO_2 at 30 ^oC within air.

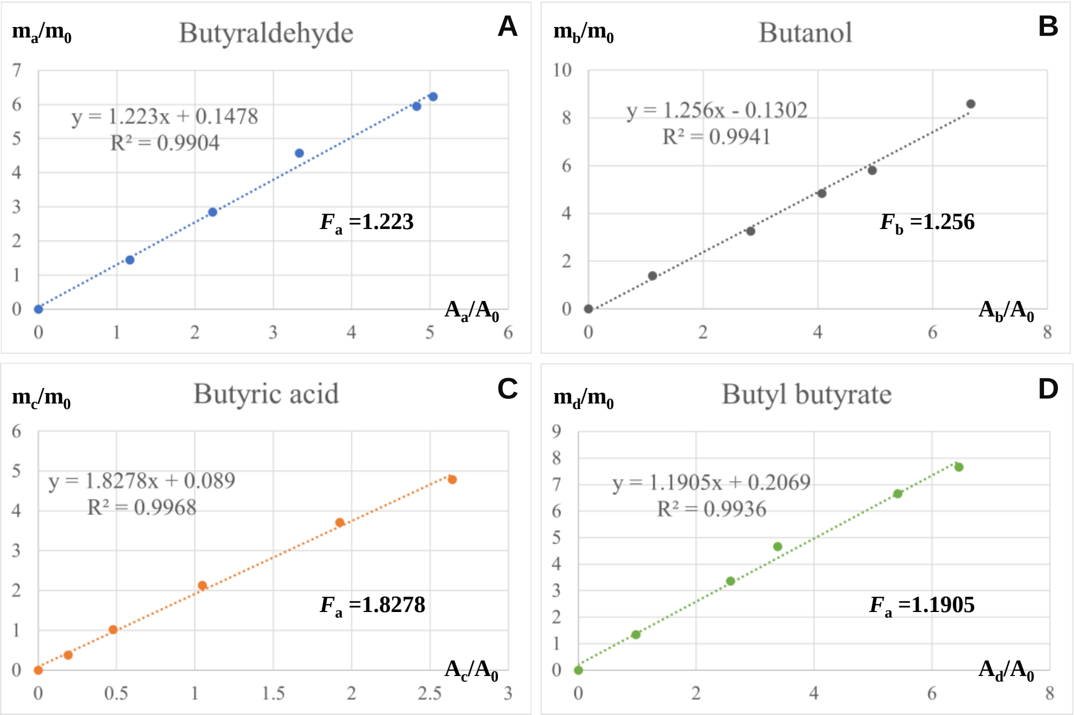

Figure S3. The GC calibration curves of butyraldehyde, butanol, butyric acid, and butyl butyrate.
